# An attention enhanced dilated bottleneck network for kidney disease classification

**DOI:** 10.1038/s41598-025-90519-w

**Published:** 2025-03-21

**Authors:** J. Jenifa Sharon, L. Jani Anbarasi

**Affiliations:** School of Computer Science and Engineering, Vellore Institute of Technology, Chennai, India

**Keywords:** Renal disease, Transfer learning models, DBAR_Net model, Pattern recognition module and bottleneck attention module, Health services, Medical imaging

## Abstract

Computer-Aided Design (CAD) techniques have been developed to assist nephrologists by optimising clinical workflows, ensuring accurate results and effectively handling extensive datasets. The proposed work introduces a Dilated Bottleneck Attention-based Renal Network (DBAR-Net) to automate the diagnosis and classification of kidney diseases like cysts, stones, and tumour. To overcome the challenges caused by complex and overlapping features, the DBAR_Net model implements a multi-feature fusion technique. Two fold convolved layer normalization blocks $\:({\text{C}\text{L}\text{N}}_{\text{b}1}$& $\:{\text{C}\text{L}\text{N}}_{\text{b}2})$ capture fine-grained detail and abstract patterns to achieve faster convergence and improved robustness. Spatially focused features and channel-wise refined features are generated through dual bottleneck attention modules $\:{(\text{A}}_{\text{b}\text{a}\text{m}1})\:\&\:{(\text{A}}_{\text{b}\text{a}\text{m}2})$ to improve the representation of convolved features by highlighting channel and spatial regions resulting enhanced interpretability and feature generalisation. Additionally, adaptive contextual features are obtained from a dilated convolved layer normalisation block $\:\left({\text{D}\text{C}\text{L}\text{N}}_{\text{b}}\right)$, which effectively captures contextual insights from semantic feature interpretation. The resulting features are fused additively and processed through a linear layer with global average pooling and layer normalization. This combination effectively reduces spatial dimensions, internal covariate shifts and improved generalization along with essential features. The proposed approach was evaluated using the CT KIDNEY DATASET that includes 8750 CT images classified into four categories: Normal, Cyst, Tumour, and Stone. Experimental results showed that $\:\text{t}\text{h}\text{e}$ improved feature detection ability enhanced the performance of DBAR_Net model attaining a F1 score as 0.98 with minimal computational complexity and optimum classification accuracy of 98.86%. The integration of these blocks resulted in precise multi-class kidney disease detection, thereby leading to the superior performance of DBAR_Net compared to other transfer learning models like VGG16, VGG19, ResNet50, EfficientNetB0, Inception V3, MobileNetV2, and Xception.

## Introduction

Kidney disease can manifest in different forms such as tumors, stones, and cysts^1^. Each type of kidney disease impacts the organ in a unique manner, exhibiting diverse causes, symptoms, and treatment approaches. Undiagnosed kidney problems can develop to kidney complications, perhaps manifesting as hematuria, pain in the abdomen, or a palpable tumor, eventually resulting in renal carcinoma. Failure to take proper care of the malignancy or if it spreads to neighboring tissues or organs can result in significant consequences. The development of kidney disease can be attributed to a range of variables, including genetic predisposition, underlying medical conditions, certain medications, and lifestyle choices such as eating patterns and hydration practices. Early detection and appropriate care are crucial for sustaining kidney function and overall renal health.

Artificial intelligence (AI)^2^ has made significant progress in the field of medical imaging, providing a wide range of applications to improve the accuracy of diagnoses, speed up procedures, and contribute to more individualized patient care identifying kidney illness using medical imaging techniques, such as Computed Tomography (CT) and Positron Emission Tomography (PET) scans, presents specific challenges that conventional approaches often fail to address. The classification and segmentation of kidney diseases are complex tasks that often require the use of medical imaging and machine learning techniques.

Conventional approaches to classifying kidney disease involve manual techniques, wherein nephrologists visually examine medical images (such as CT or PET scans)^3^ or evaluate clinical data to diagnose and classify problems with the kidneys. On the other hand, Computer-Aided Technologies (CAx)^4^ utilize automatic classification methods that rely on machine learning algorithms, particularly deep learning models, to make predictions without the need for human intervention. The utilization of deep learning techniques allows for the timely identification and classification of kidney disease by training models to autonomously classify different types of renal diseases using input data. The presented research aims to analyze and classify different types of kidney diseases, including Cyst, Stone, Tumor, and Normal conditions^5^ based on CT images. Transfer learning models^6,7^ like VGG16, VGG19, ResNet50, EfficientNetB0, Inception V3, MobileNetV2, and Xception have been used specifically for classifying renal illness. A custom framework, incorporating convolutional and other layers, has been developed to analyze different types of kidney diseases. The findings indicate superior performance compared to transfer learning models^8,9^. This approach effectively reduces the likelihood of overfitting, especially when working with limited datasets. The efficacy of transfer learning models^10^ and attention models relies on the suitability of the chosen model for the target task.

The major contributions of the proposed model are:

- The DBAR_Net model improved convergence and robustness with custom-designed dual-fold convolved layer normalization blocks $\:({\text{C}\text{L}\text{N}}_{\text{b}1}$ &$\:{\:\text{C}\text{L}\text{N}}_{\text{b}2})$. The resulting features are fed into the bottleneck attention module $\:{\:(\text{A}}_{\text{b}\text{a}\text{m}})$ which enhances convolved features by emphasizing crucial channel and spatial areas, thereby boosting interpretability and feature generalization.
- Additionally, by incorporating dilated convolved layer normalization $\:\left({\text{D}\text{C}\text{L}\text{N}}_{\text{b}}\right)$, the model captures a broader range of contextual insights, enhancing feature semantics. The resulting features undergo feature-fusion, global average pooling, and layer normalization to aggregate and reduce spatial dimensions while preserving important features, reducing internal covariate shift, and enhancing model generalization.
- The purposeful integration of these blocks aims to tackle classification challenges, resulting in enhanced feature discrimination essential for precise multi-class kidney disease detection. Their effective incorporation into DBAR_Net yields superior performance compared to VGG16, VGG19, ResNet50, EfficientNetB0, Inception V3, MobileNetV2, and Xception models.

The structure of the proposed work is given as follows: Sect. 2 presents the related studies in the field of kidney disease detection, Sect. 3 details the proposed Dilated Bottleneck Attention based Renal Network (DBAR_Net) model. Section 4 present the experimental analysis of the proposed work and Sect. 5 details the conclusion and future work.

## Related work

Han et al.^11^ performed Random Forest (RF) to analyze Corticomedullary phase (CMP), Nephrographic phase (NP), and Excretory phase (EP) in CT images resulting 90% accuracy in CMP-NP phase. This study utilized a dataset collected from histopathological reports that were based on the WHO/ISOP nuclear grading at Qilu Hospital of Shandong University. Mahmud et al.^12^ explored Res-Net50, VGG16, CNN-6, CNN-4, ResNet152, DenseNet201, InceptionV3, MobileNetV2, and DenseAUXNet201 where DenseAUXNet201 achieved high accuracy of 90.63% in KiTS21 dataset. Jhumka et al.^13^ investigated kidney disease classification using the xResNet 50 Classification model in conjunction with the SHAP (Shapely Additive exPlanations) library, achieving a classification accuracy of 97%. The dataset utilized in the study was obtained from multiple hospitals in Bangladesh and is publicly available on the Kaggle website. Chen et al.^14^ evaluated kidney disease using a combination of an Adaptive Hybridized Deep Convolutional Neural Network (AHDCNN) and Support Vector Machine (SVM) using CT images obtained from the National Institutes of Health (NIH). The AHDCNN consists of three stages: spatial max pooling, group normalization, and ReLU (Rectified Linear Unit activation function) gating integrated with linear convolution and activation functions like sigmoid, Tanh, and ReLU resulting versatile and improved features with 97.3% accuracy.

Bhandari, Mohan, et al.^15^ performed a study on the classification and segmentation of renal disorders utilizing CT scans. A lightweight customized Convolutional Neural Network (CNN) was employed to identify kidney diseases, and the classification procedure integrated Explainable Artificial Intelligence (XAI) methods including Local Interpretable Model-Agnostic Explanations (LIME) and SHAP. The model was trained using a ten-fold cross-validation approach and attained an accuracy of 98.88%. The dataset used for the identification of Kidney illness comprised 3709 cysts, 5077 normal cells, 1377 stones, and 2283 tumors obtained from Kaggle. Hadjiyski et al.^16^ performed a study on kidney cancer by analysing CT scans obtained from the TCGA-KIRC database using Deep Learning Neural Network (DLNN). The classification performance in distinguishing kidney cancer stages 1 through 4 was assessed using the Area Under the Curve (AUC) and estimated accuracy based on DLNN scores. The DLNN exhibited high accuracy in classifying the stages, achieving scores of 0.946 for Stage 1, 0.050 for Stage 2, 0.036 for Stage 3, and 0.031 for Stage 4 on the test CT scans images.

Rajkumar et al.^17^ applied two deep learning models to automatically detect and classify kidney carcinoma. The dataset, acquired from the Kaggle website, comprises 3709 images representing cysts, 5077 images of normal kidneys, 1377 images of kidney stones, and 2283 images of kidney cancers. The renal disease classification using ANN and CNN model from this among these model CNN achieved 99.6% accuracy get best performance result. Asif et al.^18^ utilized pre-trained models, including VGG19 and a naive inception module, for the detection and classification of kidney illnesses (cyst, stone, tumour) from CT images, these images are obtained from a publicly available repository called CT KIDNEY DATASET The model achieved accuracies of 96.37% and 99.25% for the VGG19 and naive inception modules, respectively.

Alzu’bi et al.^19^ performed an evaluation of VGG16 and obtained a training accuracy of 59.3% and a validation accuracy of 60%. However, ResNet50 exhibited excellent results, with a training accuracy of 97.4% and a validation accuracy of 96%. Furthermore, the CNN-6 and CNN-4 models showed remarkable results achieving training accuracies of 95.3% and 97.7%, and validation accuracies of 97% and 92%, respectively, dataset obtained from the King Abdullah University Hospital (KAUH) has been collected, consisting of 8,400 CT scan images taken from 120 adult patients who were scanned for suspected kidney masses. Hwang et al.^20^ utilized ResUNet, DenseUNet, Attention U-Net and Reversed Boundary Channel Attention Network (RBCA-Net) attaining accuracy of 77%, 79%, 81% and 82% respectively in KiTS dataset.

Hsiao et al.^21^ segmented CT images using EfficientNet-B5 as the encoder and a feature pyramid network (FPN) as the decoder in the 3D-IRCADb-01 dataset. The performance of their constructed model was evaluated using various evaluation metrics, which included a Dice score of 91.50, recall of 96.43, precision of 87.22, and an Intersection over Union score (IoU Score) of 84.42. Islam et al.^22^ performed an analysis of renal disease using advanced versions of vision transformers, including EANet, CCT, and Swin Transformers, in addition to transfer learning models like ResNet, VGG16, and Inception v3. The dataset utilized was taken from Kaggle, and attained an accuracy of 98.20% and 99.30% for VGG16 and swin transformers, respectively.

Chaudhary et al.^23^ conducted an assessment using clinical data and CT scans for two tasks: determining patient eligibility for adjuvant therapy (Task 1) and categorizing risk groups based on the American Urological Association (AUA) guidelines (Task 2). CNN was employed for imaging, CNN + MLP for a combination of imaging and clinical data, and TabNet for clinical data. Utilizing the “KNIGHT challenge” database, the categorization achieved an AUC of 0.813 in Task 1 and 0.626 in Task 2, underscoring TabNet’s potential in renal cancer care. Zhang et al.^24^ conducted a study focusing on segmenting renal diseases from CT images. They employed a Coarse to Fine (C2F) segmentation methodology, which integrated an atlas-based approach with a CNN resulting in an accuracy of 98.69% with a deviation of 8.33% in MICCAI KiTS19 dataset.

Yildirim et al.^25^ analyzed a dataset that includes CT images acquired from Philips Healthcare, Ingenuity Elite (Netherlands), and is publicly available on GitHub. The study comprised a total of 433 participants, with 278 having positive stone results and 165 categorized as normal. The XResNet-50 model which is cross-residual network analyzed this private dataset to identify stone and achieved an accuracy of 96.82%. Mehedi et al.^7^ segmented CT KIDNEY dataset using UNet and SegNet, while MobileNetV2, VGG16, and InceptionV3 were utilized for classification. The UNet model achieved a 99.48% accuracy, while the model presented an accuracy of 97%, 96%, 97%, 99% and 97%. Consequently, the VGG16 model emerged as the model with the greatest accuracy in their analysis.

Jason et al.^6^ performed Mask Region Convolutional Neural Network (Mask-RCNN) and U-Net attaining test Dice scores as 94.9 in KiTS19 dataset. Da Cruz et al.^26^ utilized AlexNet for segmentation, achieving an average Dice coefficient of 96.33%, a Jaccard index of 93.02%, sensitivity of 97.42%, specificity of 99.94%, and accuracy of 99.92%. Türk et al.^27^ evaluated ally V-Net, Fusion V-Net, ET-Net, and Hybrid V-Net achieving an accuracy of 92.1%. Fuzhe et al.^28^ analyzed renal illness by employing a Heterogeneous Modified Artificial Neural Network (HMANN) that integrated SVM and Machine Learning algorithms-Backpropagation (MLP-BP) to improve the classification of ultrasound images. The model attained a classification accuracy of 97.50% for the ultrasound images obtained from the UC Irvine Machine Learning Repository.

Elmahy et al.^29^ studied the evaluation of the Cancer Genome Atlas (TCGA) dataset, with a specific emphasis on TCGA-KIRCA (Kidney Renal Clear Cell Carcinoma). The research was conducted using Graph Convolution Networks (GCN), Random Forest (RF), and SVM. The GCN accurately classified KIRC with a precision of 73%. The accuracy of the model improved to 75% by using a weighted GCN with a correlation value of 0.5 or higher. Significant enhancements were noted when using a threshold higher than 0.7, resulting in an accuracy rate of 77%. Also, a two-weighted GCN along with graph pooling, and correlation value cut-off to be more than 0.7, resulted in the best results achieving an accuracy of 82%.

Umirzakova, Sabina, et al.^30^ proposed Deep Residual Feature Distillation Channel Attention Network (DRFDCAN) which enhances edge and texture details using high-frequency feature extraction for faster processing. The model attained SSIM (Structural Similarity Index Measure) as 85.12%, 94.68%, 94.78%, and 89.86% for OASIS, BraTS, ACDC and COVID datasets respectively. Various approaches for kidney disease classification have been proposed^31–33^, including the modified metaheuristic algorithm (mINFO) and radiomics-based methods. Pseudo-mask guided feature aggregation (PG-FANet) and breast tumor classification system^34,35^, providing enhanced classification performance in experimental results. Additionally, advanced models have been used to analyze various diseases, such as skin lesions and esophageal tumors, achieving improved segmentation precision and demonstrating efficacy across multiple datasets^36–38^. Table 1 provides an overview of the methodology, dataset, and results related to the detection and classification of kidney disease.

**Table 1:** Comparative analysis of existing literature on kidney disease classification.

Ref	Dataset	Image count	Method	Advantage and disadvantage	Accuracy (%)
^6^	KiTS 19 dataset	210 abdominal CT images	Transfer learning	Improve performance and robustness	Dice 94.9
^7^	CT KIDNEY	12,446 unique CT	Transfer learning	Lack of insights regarding the interpretability	99.48
^11^	Qilu Hospital Shandong University	51patients	Machine learning	Limited sensitivity due internal cross-validation	90
^12^	KiTS21 dataset	544 CT images	Transfer learning	Limited dataset	90.63
^13^	Kaggle PACS	12,446 unique CT	Deep learning	Not able to distinguish the tumor due to noise	97
^14^	National Institutes of Health	10,594 CT scans	Modified CNN	Optimal object recognition	97
^15^	Kaggle PACS	12,446 unique CT	CNN	Interpretation of SHAP results	98
^16^	TCGA-KIRC	4,235 images	Deep learning	Reduced misclassifications	91
^17^	Kaggle PACS	12,446 unique CT	Deep learning	Overfitting and missing data	97
^18^	CT KIDNEY	12,446 unique CT	Transfer learning	Versatility in model utilization	99
^19^	Kaggle PACS	12,446 unique CT	CNN and ANN	Imbalanced dataset	99
^20^	KiTS19 Dataset	210 abdominal CT images	Transfer learning	Irregular lesion shapes	82
^22^	CT KIDNEY	12,446 unique CT	Vision transformers	Generalization and robustness.	99
^23^	KNIGHT challenge	300,100 for training and testing	CNN + MLP	Not fully leveraged for the localized	AUC: 81
^25^	Philips Healthcare, Ingenuity Elite	1799 images	Transfer learning	Visual information about stone localization	96
^26^	KiTS19 dataset	210 abdominal CT images	Deep learning	Improved accuracy	99
^27^	KiTS19 dataset	210 abdominal CT images	Transfer learning	Precise delineation	92

## Proposed methodology

**Fig. 1:**
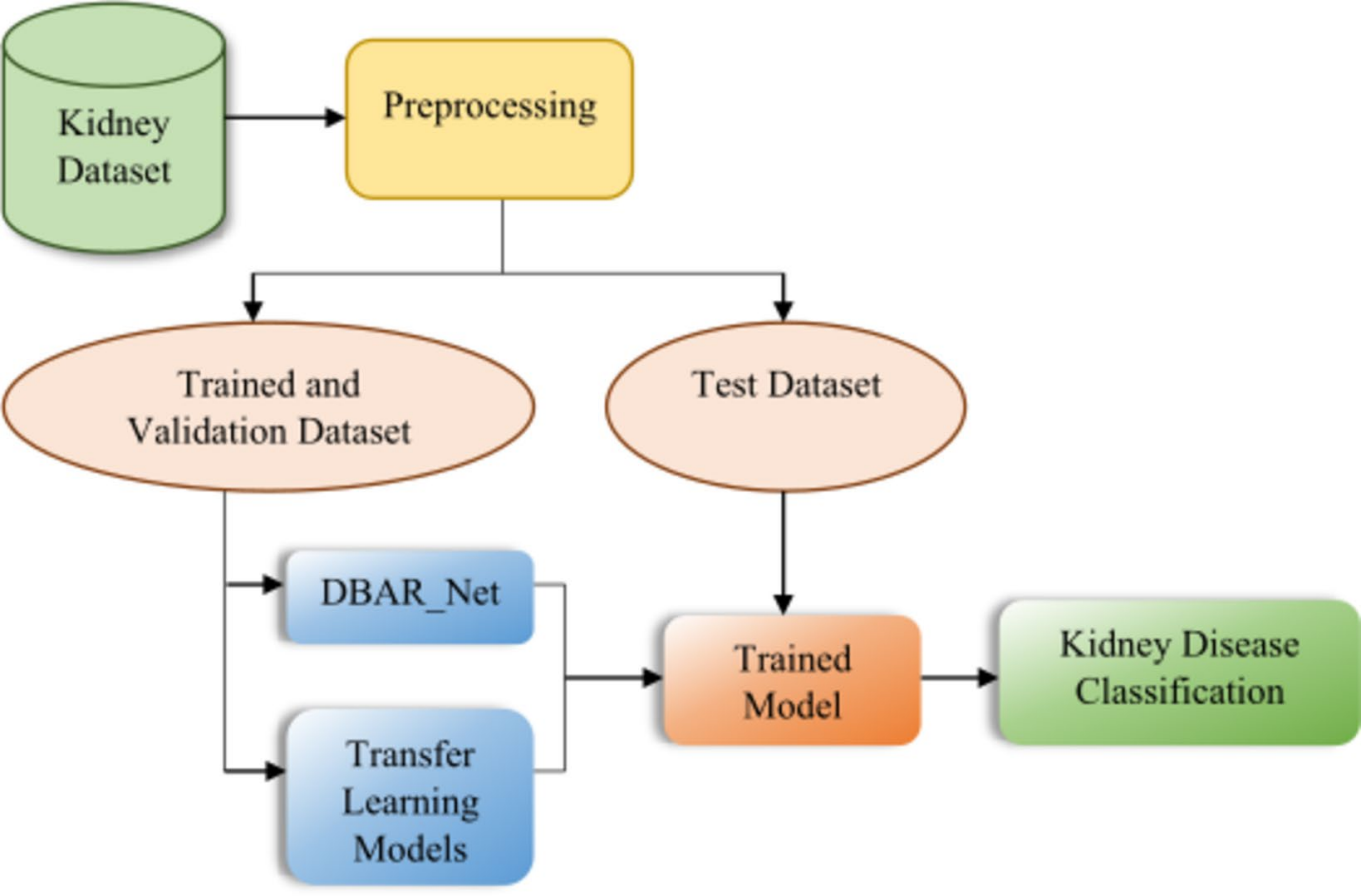
Flowchart of the proposed model.

This research provides the custom DBAR_Net model, which is an enhanced convolutional neural network designed specifically for the identification and categorization of kidney diseases through the analysis of CT images. The flow of the proposed work is given in Fig. 1.

**Fig. 2:**
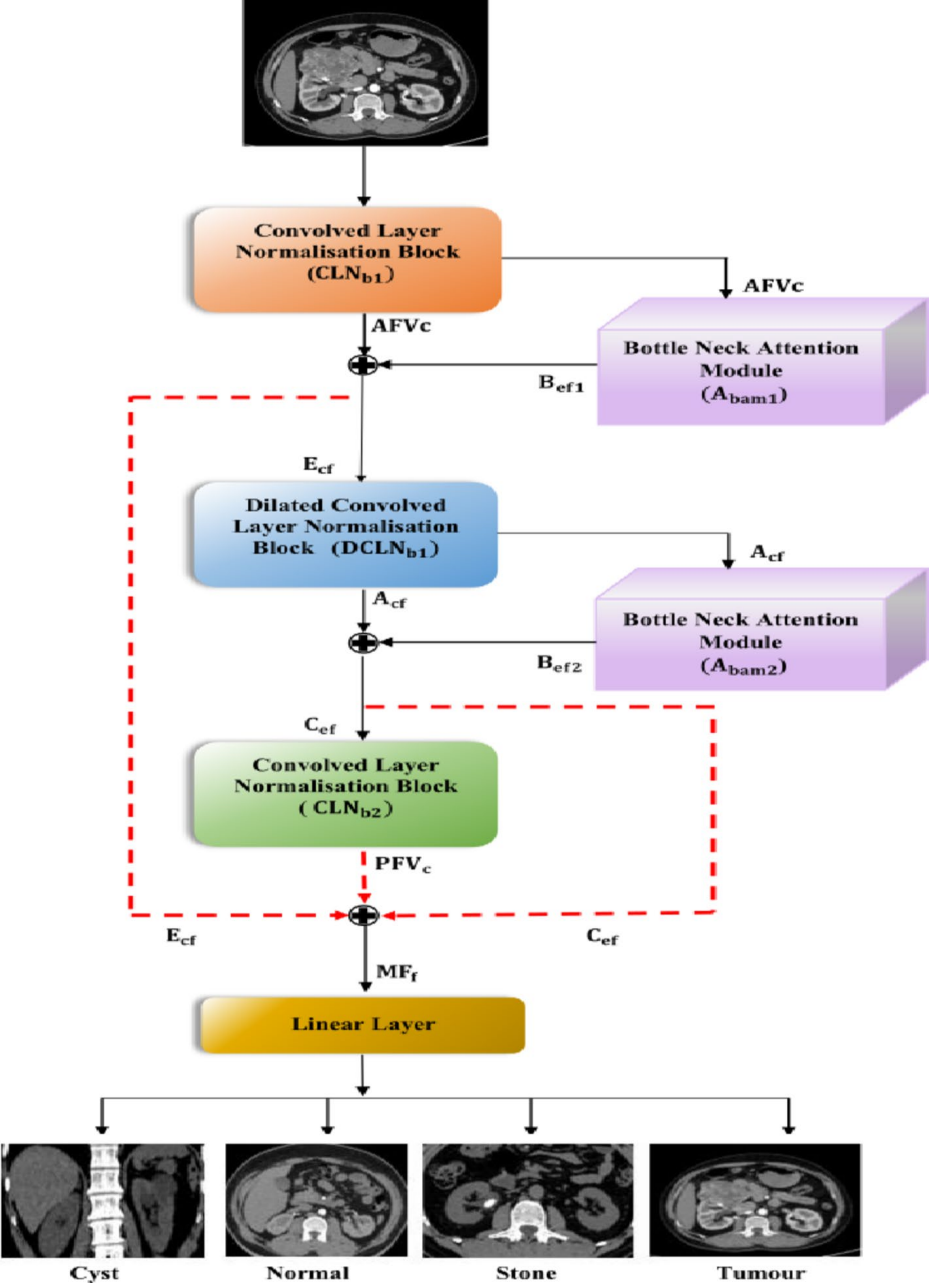
Architecture view of proposed system.

The DBAR_Net model is design by interconnecting the custom twofold convolved layer normalisation block $\:{\:\:(\text{C}\text{L}\text{N}}_{\text{b}1}\:\&\:{\text{C}\text{L}\text{N}}_{\text{b}2\:})$ along with dual bottleneck attention module $\:{\:(\text{A}}_{\text{b}\text{a}\text{m}1})\:\&\:{(\text{A}}_{\text{b}\text{a}\text{m}2})$ and dilated convolved layer normalization ($\:{\text{D}\text{C}\text{L}\text{N}}_{\text{b}})$ .The feature extracted from these blocks are fused together and finally connected to linear layer. Specifically$\:{\:\:(\text{C}\text{L}\text{N}}_{\text{b}1}\:\&\:{\text{C}\text{L}\text{N}}_{\text{b}2\:}$) is used to capture the most salient characteristics of the images and build effective functionalities between these network. These modules comprised of convolutional layers, global average pooling, and layer normalization^39^ are strategically placed to enhance features representation and normalization within the network. The bottleneck attention module is systematically placed between convolved and dilated convolved layer normalization to highlight essential elements within the input sequence. This module integrates both channel attention and spatial attention modules^20^, directing the model to focus on important feature maps within the input tensor. This configuration enables enhanced localization of characteristics within an image, ultimately improving accuracy in tasks such as object recognition. The subsequent layer in the network architecture is the linear layer which includes dense and flatten layer and all these layer is making the DBAR_Net model is seen in Fig. 2 for better prediction and classification of kidney disease.

The kidney dataset is processed by the convolved layer normalisation block$\:{\:\:(\text{C}\text{L}\text{N}}_{\text{b}1})$, which extracts the aggregated feature vector channel $\:{\:(\text{A}\text{F}\text{V}}_{\text{c}})$. The retrieved features are then fine-tuned using the bottleneck attention module $\:{(\text{A}}_{\text{b}\text{a}\text{m}1})\:$yielding refined feature maps $\:{(\text{B}}_{\text{e}\text{f}1})$. Subsequently, the $\:{\text{B}}_{\text{e}\text{f}1}$ feature passes through to the dilated convolved layer normalisation block $\:{\:(\text{D}\text{C}\text{L}\text{N}}_{\text{b}})\:$that employs dilated convolution to augment the receptive field and derive adaptive contextual features $\:{(\text{A}}_{\text{c}\text{f}}$). The contextual characteristics are further improved by the $\:{\text{A}}_{\text{b}\text{a}\text{m}2}\:\text{b}\text{l}\text{o}\text{c}\text{k}$ resulting bottleneck enhanced features $\:{(\text{B}}_{\text{e}\text{f}2}).$ This features are then optimized by the convolved layer normalisation block $\:{(\text{C}\text{L}\text{N}}_{\text{B}2})\:$resulting in the final processed channel vector features $\:{\:(\text{P}\text{F}\text{V}}_{\text{c}}$). The outputs from different block $\:{(\text{C}}_{\text{e}\text{f}})\:,\left({\text{E}}_{\text{c}\text{f}}\right)$ and$\:{\:(\text{P}\text{F}\text{V}}_{\text{c}}$) are integrated using feature fusion, as illustrated in the diagram. The result produces an effective feature representation termed as$\:{\:\text{M}\text{F}}_{\text{f}}$. The fused features are subjected to linear layer that classifies them into four categories of kidney disease: cyst, normal, stone, and tumour.

### Convolved layer normalisation block ($\:{\text{C}\text{L}\text{N}}_{\text{b}}$)

**Fig. 3:**
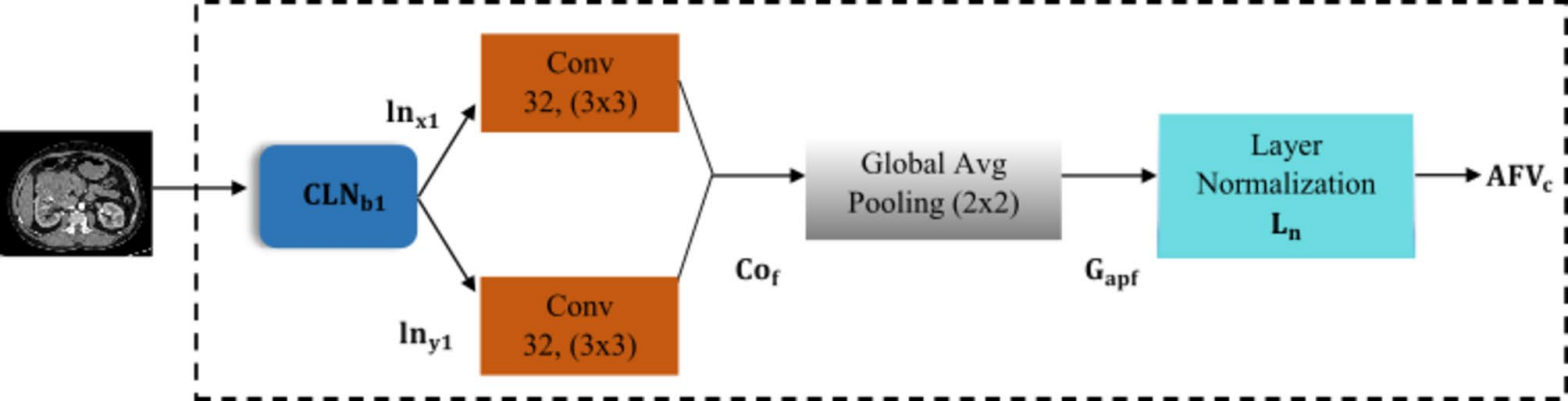
Schematic representation of convolved layer normalization block.

The input kidney dataset is fed to the convolved layer normalisation block$\:{\:(\text{C}\text{L}\text{N}}_{\text{b}1})$ is show in Fig. 3 that stabilizes the learning process to effectively categorise four classes using layered normalisation technique and ReLU. The input is fed to two convolutional layers where first layer captures low-level features, and subsequent layers learn to combine these features to form more complex, higher-level representations resulting in hierarchical receptive global features about each data. The non-linearity features $\:{\text{l}\text{n}}_{\text{x}1,\text{y}1}^{\text{l}\text{a}}\:$are generated by summing up the weighted filter $\:{\text{f}}_{\text{i},\text{j}}\:\:$of size $\:\text{m}\:\text{x}\:\text{m}$ of input components $\:\text{I}\text{n}$ from the previous layer as shown in Eq. (1).

1 $$\:{\text{C}\text{o}}_{\text{f}}{=\text{l}\text{n}}_{\text{x}1,\text{y}1}^{\text{l}\text{a}}={\upsigma\:}\sum\:_{\text{i}=1}^{\text{m}}\sum\:_{\text{j}=1}^{\text{m}}{\text{f}}_{\text{i},\text{j}}{\text{I}\text{n}}_{\text{n}\left(\text{x}+\text{i},\text{y}+\text{j}\right)}^{\text{l}\text{a}-1}$$

These resultant nonlinear complex features are fed to global average pooling layer that translates invariance by selecting the maximum value within each pooling region thereby improving its robustness. The convolutional feature map $\:{(\text{C}\text{o}}_{\text{f}})$ with spatial feature information size $\:\text{x},\text{y},\text{z}$, kernel size $\:\text{k}\text{x}\text{k}$, and stride$\:{\:\text{S}}_{\text{t}}$ as 2 results in an output feature map $\:{(\text{G}}_{\text{a}\text{p}\text{f}})$ with reduced spatial dimensions as shown in Eq. (2).

2 $$\:{\text{G}}_{\text{a}\text{p}\text{f}}=(\text{x}-\text{k}+1)/{\text{S}}_{\text{t}}\:\text{X}(y-k+1)/{\text{S}}_{\text{t}} \text{X} \:z\:$$

This resultant $\:{\text{G}}_{\text{a}\text{p}\text{f}}$ is fed to layer normalization $\:{\text{L}}_{\text{n}}$ that normalizes the activations along the feature direction instead of mini-batch direction. The layer normalized features $\:{\text{L}}_{\text{n}}$ transforms the feature maintaining the mean and standard deviation close to 0 and 1 respectively. The layered normalized feature is computed using mean and standard deviation as shown in Eqs. (3) and (4), where $\:{\upepsilon\:}$ is the constant, $\:{\text{x}}_{\text{i}}$ is the set of pixels.

3 $$\:\:\:\text{M}\text{e}\text{a}\text{n}\left({{\upmu\:}}_{\text{i}}\right)=\:\frac{1}{\text{i}}{\sum\:}_{\text{i}\in\:{\text{x}}_{\text{i}}}{\text{G}}_{\text{a}\text{p}\text{f}}$$

4 $$\:\text{S}\text{t}\text{a}\text{n}\text{d}\text{a}\text{r}\text{d}\:\text{d}\text{e}\text{v}\text{i}\text{a}\text{t}\text{i}\text{o}\text{n}\left({{\upsigma\:}}_{\text{i}}\right)=\:\sqrt{\frac{1}{\text{i}}}{{\sum\:}_{\text{i}\in\:{\text{x}}_{\text{i}}}{(\text{G}}_{\text{a}\text{p}\text{f}}-{{\upmu\:}}_{\text{i}})}^{2}+\:{\upepsilon\:}$$

Layer normalization denoted as $$\:{\text{L}}_{\text{n}}$$ is achieved by incorporating learnable scaling and shifting $$\:({\upalpha\:}\:\text{a}\text{n}\text{d}$$$$\:{\upbeta\:})$$ parameters, which facilities feature normalization. This process is intended to improve comparability among coefficients or parameters associated with each feature. There by enhancing interpretability within the model. Mathematically, the layer normalization is expressed as shown in Eq. (5).

5 $$\:{\text{L}}_{\text{n}}=\:{{\upsigma\:}}_{\text{i}}\:\frac{{\text{G}}_{\text{a}\text{p}\text{f}}-\:{\upmu\:}}{{\upsigma\:}}+\:{\upbeta\:}$$

Here, $\:{\text{G}}_{\text{a}\text{p}\text{f}}$ represent the input feature, $\:{\upmu\:}$ is the mean and $\:{\upsigma\:}$ is the standard deviation, ensuring that the input is normalized. The learnable parameter allows the model to adapt and potentially reverse normalization. Following layer normalization, the element wise application of the ReLU activation function is used and denoted as $\:{\varnothing}(\cdot\:).$ This approach regulates training by reducing internal covariate shift and non- linearity using thresholding the values, resulting is the final output aggregated channel vector feature$\:{\:\text{A}\text{F}\text{V}}_{\text{c}}$.

6 $$\:\:\:{\text{A}\text{F}\text{V}}_{\text{c}}=\:{\varnothing}\left({\text{L}}_{\text{n}}\right)$$

The aggregated feature vector channel$\:{\:(\text{A}\text{F}\text{V}}_{\text{c}}$) contain the activated values, introducing non-linearity to the model. This final output feature captures the transformed and normalized representation of input is subsequently subjected to bottleneck attention module $\:{(\text{A}}_{\text{b}\text{a}\text{m}})$.

### Bottleneck attention module (${\mathbf{A}}_{\mathbf{b}\mathbf{a}\mathbf{m}}$)

**Fig. 4:**
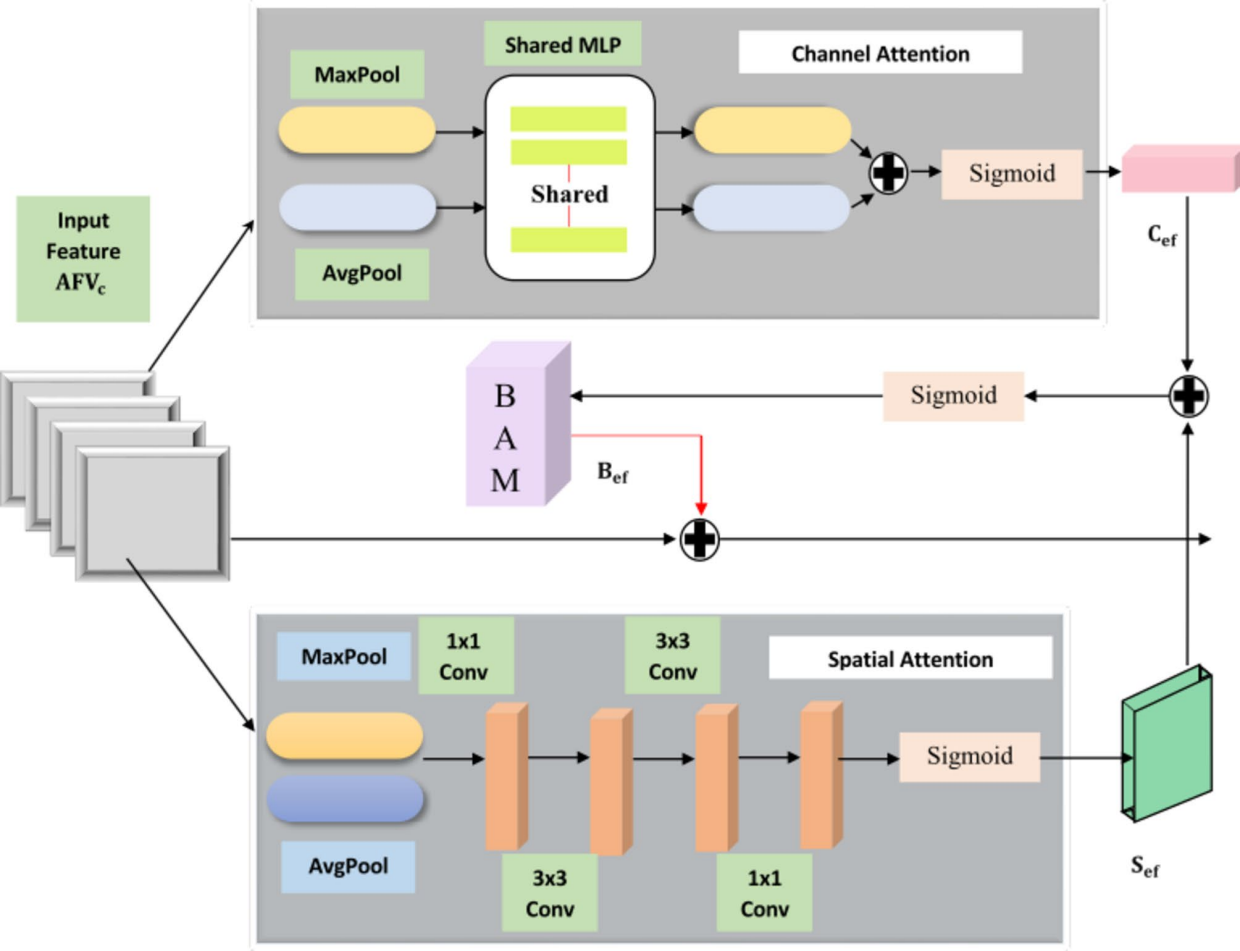
The structure of the bottleneck attention module.

The generated aggregated layered feature vector$\:{\:\text{A}\text{F}\text{V}}_{\text{c}}\:$ by the convolved layer normalisation block is fed to a comprehensive processing through a bottleneck attention module, represented as $\:{(\:\text{A}}_{\text{b}\text{a}\text{m}1})\:$is shows in Fig. 4. The channel $\:\left({\text{A}}_{\text{c}\text{h}}\right)$ and spatial attention$\:\:\left({\text{A}}_{\text{s}\text{p}}\right)$ mechanisms are integrated to enhance the attention capabilities and interpretability of the proposed model. The spatial attention $\:{(\text{A}}_{\text{s}\text{p}})$ enhances specific feature with in convolutional spatial layer by incorporating appropriate filters with a sigmoid activation function. An element wise multiplication is performed with aggregated features $\:{\:(\text{A}\text{F}\text{V}}_{\text{c}})$ emphasising spatial local feature$\:\:{(\text{S}}_{\text{e}\text{f}})$ patterns to effectively identify the types of kidney diseases. The spatial recalibrated feature $\:{(\text{S}}_{\text{e}\text{f}}$) are obtained by weighted summation with the convolved feature as given in Eq. (7).

7 $$\:{\text{S}}_{\text{e}\text{f}}=\:{\:\text{A}\text{F}\text{V}}_{\text{c}}\:{\odot}\:{\:\text{A}}_{\text{s}\text{p}}$$

In parallel, the channel-wise information $\:{\text{A}\text{F}\text{V}}_{\text{c}}$ is fed into the channel attention mechanism $\:{(\text{A}}_{\text{c}\text{h}})$ for scaling each channel by its attention weight. This emphasizes the generation of enhanced global feature patterns by computing a set of weights $\:\text{w}$ through a fully connected layer with specific filters and a sigmoid activation function. The bottleneck attention module results enhanced features $\:{(\text{B}}_{\text{e}\text{f}1})$ by integrating spatial and channel characteristics, enhancing its capacity to discern between relevant and irrelevant features. This enhancement contributes to its improved generalization across various disease types, as illustrated in the Eqs. (8) and (9).

8 $$\:{\text{C}}_{\text{e}\text{f}}=\:{\:\text{A}\text{F}\text{V}}_{\text{c}}\:{\odot}\:{\:\text{A}}_{\text{c}\text{h}\text{w}}$$

9 $$\:\:{\text{B}}_{\text{e}\text{f}1}=\:{\text{C}}_{\text{e}\text{f}}\:{\oplus}{\:\:\text{S}}_{\text{e}\text{f}}$$

**Fig. 5:**
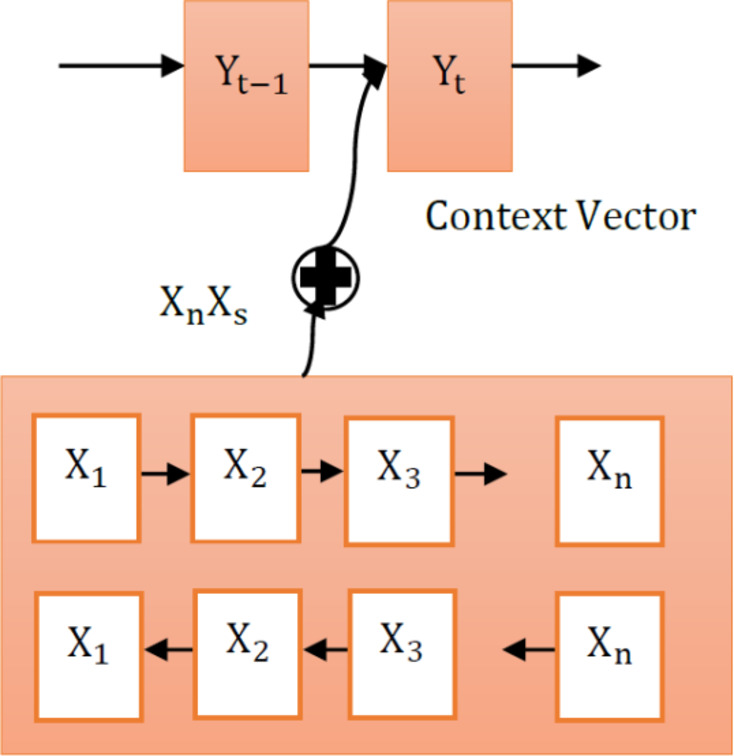
Workflow of the attention mechanism.

The bottleneck enhanced feature $\:{\text{B}}_{\text{e}\text{f}1}$ is subsequently concatenate with $\:{\:(\text{A}\text{F}\text{V}}_{\text{c}}$) resulting enhanced convolved feature$\:\:\left({\text{E}}_{\text{c}\text{f}}\right)$ as expressed in Eq. (10). This operation merges the enriched features obtained from the attention mechanism with the aggregated feature vector channel, enhancing informative channels and critical regions within the intermediate features, thereby boosting performance. Figure 5 illustrates the explanation of the attention mechanism.

10 $$\:{\text{E}}_{\text{c}\text{f}}=\:{\:\text{A}\text{F}\text{V}}_{\text{c}\:}{\oplus}\:{\text{B}}_{\text{e}\text{f}1}$$

### Dilated convolved layer normalisation block $\:{(\text{D}\text{C}\text{L}\text{N}}_{\text{b}})$

**Fig. 6:**
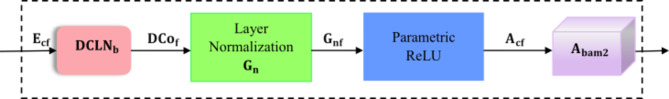
Design of the dilated convolved layer normalization block.

The attention enhanced feature $\:{\text{E}}_{\text{c}\text{f}}$ undergoes dilated convolved layer normalization $\:{\:(\text{D}\text{C}\text{L}\text{N}}_{\text{b}})$ is seen in Fig. 6 with learnable parameters to expand the model’s performance while preserving spatial resolution thus capturing extensive contextual information form the input feature. The resulting output feature map, labelled $\:\left({\text{D}\text{C}\text{o}}_{\text{f}}\right)$, is shown in Eq. (11), where$\:\:\text{i},\text{j}\:$ denote the spatial dimension of the feature map $\:{(\text{E}}_{\text{c}\text{f}})\:$, while $\:\text{k}$ represents the kernel size$\:,\:{\:\text{S}}_{\text{t}}$ refers the stride, $\:\text{z}$ is the number of output channels ,$\:\:\text{d}$ stands for the dilation rate, and $\:\text{m}$ indicates the filter size.

11 $$\:{\text{D}\text{C}\text{o}}_{\text{f}}=\left(\frac{\text{i}-\text{k}+\left(\text{d}\cdot\:\left(\text{m}-1\right)\right)}{{\text{S}}_{\text{t}}}+1\right)\text{X}\left(\frac{\text{j}-\text{k}+\left(\text{d}\cdot\:\left(\text{m}-1\right)\right)}{{\text{S}}_{\text{t}}}+1\right)\text{X}\:\text{z}$$

The resulting dilated feature maps are fed through group normalization along the feature dimension which enhances the networks generalization capability by organizing activations into distinct subsets. Group normalization $\:\left({\text{G}}_{\text{n}\text{f}}\right)\:$aids in adapting and learning intricate patterns, thereby improving the overall performance by computing the mean (µ) and standard deviation (σ) within activation groups as shown in Eq. (12). Parameters γ and β are employed for scaling and shifting the normalized activations, ensuring stable training and improved generalization. These adjustments allow for more stable training and promote better generalization, ultimately contributing to enhanced model performance.

12 $$\:{\text{G}}_{\text{n}\text{f}}=\:{\upgamma\:}\:\frac{{\text{D}\text{C}\text{o}}_{\text{f}}-\:{\upmu\:}}{{{\upsigma\:}}_{\text{G}\text{n}}}+\:{\upbeta\:}$$

The learnable parameter $\:\left({\upalpha\:}\right)$ introduced by the parametric ReLU activation function adaptively adjusts the activation threshold during training allowing the network to capture complex patterns and variations in the data more effectively to generate adaptive contextual features $\:{(\text{A}}_{\text{c}\text{f}}$) map that maintains spatial dimension and depicts the network’s activation subsequently to normalization and activation process, as shown in Eq. (13).

The resultant output $\:{\text{A}}_{\text{c}\text{f}}\:$is fed into bottleneck attention module$\:{\:\:(\text{A}}_{\text{b}\text{a}\text{m}2})$ to selectively amplify crucial features while suppressing irrelevant ones, thus producing bottleneck enhanced features $\:{(\text{B}}_{\text{e}\text{f}2})$. Subsequently, the $\:{\text{B}}_{\text{e}\text{f}2}$ are concatenated with contextual feature $\:{(\text{A}}_{\text{c}\text{f}})$. The resulting concatenate enhanced feature $\:\left({\text{C}}_{\text{e}\text{f}}\right)$ combines both the initial features and the refined representations obtained through attention mechanisms is show in Eq. (14). Through this integration, a more complex and precise feature representation, leading to enhanced model interpretability and performance across various tasks.

13 $$\:{\text{A}}_{\text{c}\text{f}}=\:{\text{G}}_{\text{n}\text{f}}\:{\odot}\:\:({\text{G}}_{\text{n}\text{f}\:}\:{\odot}\:{\upalpha\:})$$

14 $$\:{\text{C}}_{\text{e}\text{f}}=\:{\text{A}}_{\text{c}\text{f}}\oplus\:{\text{B}}_{\text{e}\text{f}2}$$

These $\:{\text{C}}_{\text{e}\text{f}}$ are then passed to the convolved layer normalisation block $\:{(\text{C}\text{L}\text{N}}_{\text{B}2})$ that has similar structure as$\:{\:\:(\text{C}\text{L}\text{N}}_{\text{b}1})$ for further processing. The input features, comprising both $\:{(\text{C}}_{\text{e}\text{f}}\:)$ and refined representations from attention mechanisms^40^, undergo convolution to extract higher-level features. Subsequently, global average pooling is applied to aggregate spatial information across feature maps, reducing spatial dimensions while preserving important features. Layer normalization is then employed to stabilize training by normalizing activations within each layer, reducing internal covariate shift and enhancing model generalization and the resulting is the final output processed channel vector feature$\:{\:(\text{P}\text{F}\text{V}}_{\text{c}}$).

### Multi-feature fusion

The features from $\:{(\text{E}}_{\text{c}\text{f}}),\:\:{(\text{C}}_{\text{e}\text{f}})\:\text{a}\text{n}\text{d}\:{(\text{P}\text{F}\text{V}}_{\text{c}})\:$are fused together to create the fused feature ensemble into a unified representation. Equation (15) represents the computation of the output feature $\:{\:(\text{F}}_{\text{f}1\:})$. Each element of the feature map $\:{(\text{E}}_{\text{c}\text{f}})\:$is multiplied by a corresponding weight $\:{\text{w}}_{\text{i}}\:$, where $\:\text{i}$ ranges from 1 to $\:\text{n}$. These weighted elements are then aggregated by summation, resulting in a single scalar value representing $\:{\:(\text{F}}_{\text{f}1\:})$​.

15 $$\:\:\:\:\:\:\:\:\:\:\:\:\:\:\:\:\:\:{\text{F}}_{\text{f}1}\:=\:\sum\:_{\text{i}=1}^{\text{n}}{\text{w}}_{\text{i}}\:\cdot\:\:{\text{E}}_{\text{c}\text{f}}\:\:\:\:\:\:\:\:\:\:\:\:\:\:\:\:\:\:\:\:\:\:\:\:$$

Similarly, Eq. (16) calculates the output feature$\:{\:(\text{F}}_{\text{f}2})$. It follows the same principle as Eq. (15), with each element of the feature map $\:{\text{C}}_{\text{e}\text{f}}\:$being multiplied by a corresponding weight$\:{\:\:\text{w}}_{\text{i}}$ and the weighted elements are summed up to produce$\:{\:\:(\text{F}}_{\text{f}2})$.

16 $$\:{\:\:\:\:\text{F}}_{\text{f}2}\:=\:\sum\:_{\text{i}=1}^{\text{n}}{\text{w}}_{\text{i}}\:\cdot\:{\text{C}}_{\text{e}\text{f}}$$

Equation (17) describes the computation of the output feature$\:{\:\:(\text{F}}_{\text{f}3})$. Again, each element of the feature map $\:{\:(\text{P}\text{F}\text{V}}_{\text{c}})$ is multiplied by a corresponding weight$\:{\:\:\text{w}}_{\text{i}}\:$ and the resulting products are summed to yield $\:{\:(\text{F}}_{\text{f}3})$​.

17 $$\:{\:\:\:\:\:\:\:\:\:\:\:\:\:\:\:\:\:\:\text{F}}_{\text{f}3}\:=\:\sum\:_{\text{i}=1}^{\text{n}}{\text{w}}_{\text{i}}\:\cdot\:\:{\text{P}\text{F}\text{V}}_{\text{c}}$$

After the computation describe in Eqs. (15), (16) and (17) the resulting features $\:{(\text{F}}_{\text{f}1}),\:{\:\:(\text{F}}_{\text{f}2})\:\text{a}\text{n}\text{d}\:{\:(\:\text{F}}_{\text{f}3})$ are concatenated. The feature map is stacked horizontally to solve complex non-linear problem, allowing the network to capture intricate patterns and correlations across multiple layers to extract distinctive features as shown in Eq. (18). This process aims to enhance the identification accuracy for different type of kidney disease.

18 $$\:{\:\:\:\:\text{M}\text{F}}_{\text{f}}\:=\:{\:[\:\text{F}}_{\text{f}1},\:{\:\:\text{F}}_{\text{f}2}\:,{\:\text{F}}_{\text{f}3}]\:$$

The linear layer with a ReLU activation function processes the fusion of multiple features, aiming to reduce the dimensionality of the combined feature set. This reduction enhances computational efficiency and mitigates the challenges posed by high-dimensional data. The combined feature, denoted as $\:{\:(\text{M}\text{F}}_{\text{f}})$undergoes weighting and summation along with a bias term within the linear layer additionally, learning rate and dropout technique is used to optimize the performance and prevent overfitting. Subsequently, the ReLU activation function is applied to introducing non-linearity to the model. DBAR_Net is effectively identifying various types of kidney diseases by extracting meaningful information from the multi-feature inputs.

## Experimental result and discussion

This section discusses several aspects of the analysis process, including collecting data, hyper parameter tuning^21^, model training and validation, visual interpretation, and the overall effectiveness of the proposed DBAR_Net architecture. The section systematically examines the statistical analyses of the DBAR_Net model, clarifies the results obtained from the conducted tests and presents a comprehensive comparison between various methodologies and models.

### Dataset description

**Fig. 7:**
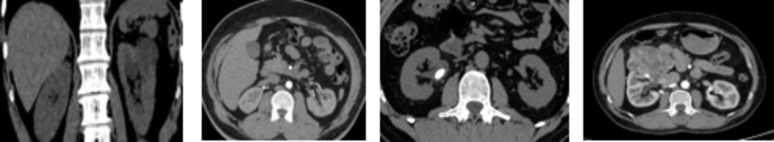
Original sample image of CT scans from the kidney disease dataset.

The dataset, which was collected from the CT KIDNEY DATASET (https://www.kaggle.com/datasets/nazmul0087/ct-kidney-dataset-normal-cyst-tumor-and-stone) collection and available to the public, consists of CT images acquired from persons diagnosed with kidney diseases in multiple hospitals throughout Bangladesh^22^. In this study 8570 distinct CT scan images taken from the dataset, including kidney cysts, stones, tumours, and normal kidneys. Coronal and axial slices were selected based on the methodology for measuring the whole abdomen and urogram, from both contrast and non-contrast testing. There are precisely 2500 images displaying kidney cysts, 1370 images showing kidneys affected by stones, and 2200 images displaying kidneys affected by tumours. The data collection consists of images with a pixel resolution of 705 × 569, with both the horizontal and vertical dimensions set at 96 × 96 dots per inch (dpi) is shown in Fig. 7.

### Experimental setup and system configuration

The model explained in this research was trained and evaluated using the Anaconda environment and Jupyter Notebook. The OpenCV library was utilized to implement data augmentation, while the Tensor Flow frameworks in Python were employed to develop the model. The system configuration used for these tasks includes an Intel(R) Core(TM) i5-6200U CPU @ 2.30 GHz 2.40 GHz processor, Intel® HD Graphics 520, 8.00 GB RAM, and an AMD Radeon (TM) R5 M335 4GB graphics card.

### Data augmentation

The dataset is subjected to data augmentation techniques with the goal of improving the model’s ability to generalize and execute effectively especially for training and testing sets. Data augmentation techniques^41^ are applied during the training phase to enhance the diversity of the training dataset. The input image is scaled down by a factor of 1/255, achieved through dividing each pixel value by 255. The method involves normalising the pixel values of an image by scaling them proportionally to fit inside the range of [0,1]. Next, a shear transformation is applied, resulting in a 20% tilt of the images, so increasing the variety of the collection. Random zooming uses pixels to allow images to be enlarged or reduced by a maximum of 20%, so introducing more variability is seen in Table 2. In addition, the implementation includes horizontal flipping, which randomly mirrors images. Rescaling is conducted on the testing dataset in order to maintain the original image parameters.

**Table 2:** Augmented sample image of CT scans for kidney disease dataset.

Augmented dataset				

### Hyper parameter tuning

The hyper parameter tuning factors is being frequently used to train neural networks for tasks including image categorization. The input image has dimensions of 705 pixels in width and 569 pixels in height, with three channels correlating to the red, green, and blue colours in an RGB image. In order to prevent exceeding the optimal values, a learning rate of 0.001 is utilised to determine the size of the modification made to the model weight during the training process. Epochs^17^ represent the outcome of a singular and complete iteration across the entire dataset. The suggested model utilises an epoch size of 35 for training the dataset, enabling the model to converge. Convergence refers to the incremental optimisation of weights to better align with the training data. The model parameter^24^ will be updated according to the average gradient computation calculated using a batch size of 30, where each iteration will handle 30 samples. The batch size is determined by the variables $\:{\text{T}}_{\text{s}}$, which represents the total number of samples and $\:{\text{N}}_{\text{E}}$, which represents the number of batches per epoch, as indicated in Eq. (19). Hyper parameters are crucial for effectively training and evaluating a model, ultimately leading to optimal model performance.

19 $$\:\:\text{B}\text{a}\text{t}\text{c}\text{h}\:\text{S}\text{i}\text{z}\text{e}=\:\frac{\:{\text{T}}_{\text{s}}}{\:{\text{N}}_{\text{E}}}$$

### Model training and validation

The training and validation processes were performed on both transfer learning models^42^ and the DBAR_Net model, using the same hyper parameter tuning, to classify kidney illnesses into four classes. The entire dataset was divided into training and validation sets using a ratio of 80:20, comprising a total of 8,750 CT scan images. Out of these, 6,856 were designated for training purposes, while 1,714 were reserved for testing. Table 3 displays the training loss and accuracy, as well as the validation loss and accuracy for transfer learning models and the proposed DBAR_Net model. These metrics are important indicators used to evaluate the success of a model during training^41^. They measure the difference between the model’s predictions and the actual target values.

**Table 3:** Training and validation curve of DBAR_Net and transfer learning model.

Model	Training/validation loss and accuracy	
VGG16		
VGG19		
ResNet50		
Xception		
MobileNet		
InceptionV3		
EfficientNetB0		
Proposed DBAR_Net		

The training and validation performance^23^ of loss is taken as training loss (L_train) is a define as the average loss over the all the training samples and validation loss (L_val) is indicate as the average loss over all validation samples. Here, $\:{\text{N}}_{\text{t}\text{r}\text{a}\text{i}\text{n}}$ and $\:{\text{N}}_{\text{v}\text{a}\text{l}}$ are the number of sample in the training and validation sets, respectively. $\:{\text{y}}_{\text{i}},\text{t}\text{r}\text{u}\text{e}$ is the true target for the, $\:\text{i}$-th sample, $\:{\text{y}}_{\text{i}},\text{p}\text{r}\text{e}\text{d}$ is the predicated target is shown in the Eqs. (20) and (21).

20 $$\:\text{T}\text{r}\text{a}\text{n}\text{i}\text{n}\text{g}\:\text{L}\text{o}\text{s}\text{s}:{\text{L}}_{\text{t}\text{r}\text{a}\text{i}\text{n}}=\:\frac{1}{{\text{N}}_{\text{t}\text{r}\text{a}\text{i}\text{n}}}{\sum\:}_{\text{i}=1}^{{\text{N}}_{\text{t}\text{r}\text{a}\text{i}\text{n}}}\text{l}\text{o}\text{s}\text{s}({\text{y}}_{\text{i}},\text{t}\text{r}\text{u}\text{e},{\text{y}}_{\text{i}},\text{p}\text{r}\text{e}\text{d})$$

21 $$\:\text{V}\text{a}\text{l}\text{i}\text{d}\text{a}\text{t}\text{i}\text{o}\text{n}\:\text{L}\text{o}\text{s}\text{s}:{\text{L}}_{\text{v}\text{a}\text{l}}=\:\frac{1}{{\text{N}}_{\text{v}\text{a}\text{l}}}{\sum\:}_{\text{i}=1}^{{\text{N}}_{\text{v}\text{a}\text{l}}}\text{l}\text{o}\text{s}\text{s}({\text{y}}_{\text{i}},\text{t}\text{r}\text{u}\text{e},{\text{y}}_{\text{i}},\text{p}\text{r}\text{e}\text{d})$$

The training and validation performance of accuracy is taken as training accuracy (A_train) as the percentage of correctly classified sample in the training set and validation accuracy (A_val) is the percentage of correctly classified sample in the validation set. Here, the number of correctly classified samples is the sum of samples for which $\:{\text{y}}_{\text{i}},\text{t}\text{r}\text{u}\text{e}$ is equal to$\:{\:\text{y}}_{\text{i}},\text{p}\text{r}\text{e}\text{d}$ explain in Eqs. (22) and (23).

22 $$\:\text{T}\text{r}\text{a}\text{n}\text{i}\text{n}\text{g}\:\text{A}\text{c}\text{c}\text{u}\text{r}\text{a}\text{c}\text{y}:{\text{A}}_{\text{t}\text{r}\text{a}\text{i}\text{n}}=\:\frac{\text{N}\text{u}\text{m}\text{b}\text{e}\text{r}\:\text{o}\text{f}\:\text{c}\text{o}\text{r}\text{e}\text{c}\text{t}\text{l}\text{y}\:\text{c}\text{l}\text{a}\text{s}\text{s}\text{i}\text{f}\text{e}\text{d}\:\text{t}\text{r}\text{a}\text{n}\text{i}\text{n}\text{g}\:\text{s}\text{a}\text{m}\text{p}\text{l}\text{e}\text{s}}{{\text{N}}_{\text{t}\text{r}\text{a}\text{i}\text{n}}}$$

23 $$\:\text{V}\text{a}\text{l}\text{i}\text{d}\text{a}\text{t}\text{i}\text{o}\text{n}\:\text{A}\text{c}\text{c}\text{u}\text{r}\text{a}\text{c}\text{y}:{\text{A}}_{\text{v}\text{a}\text{l}}=\:\frac{\text{N}\text{u}\text{m}\text{b}\text{e}\text{r}\:\text{o}\text{f}\:\text{c}\text{o}\text{r}\text{e}\text{c}\text{t}\text{l}\text{y}\:\text{c}\text{l}\text{a}\text{s}\text{s}\text{i}\text{f}\text{e}\text{d}\:\text{t}\text{r}\text{a}\text{n}\text{i}\text{n}\text{g}\:\text{s}\text{a}\text{m}\text{p}\text{l}\text{e}\text{s}}{{\text{N}}_{\text{v}\text{a}\text{l}}}$$

### Visually interpreted features of the trained model

**Fig. 8:**
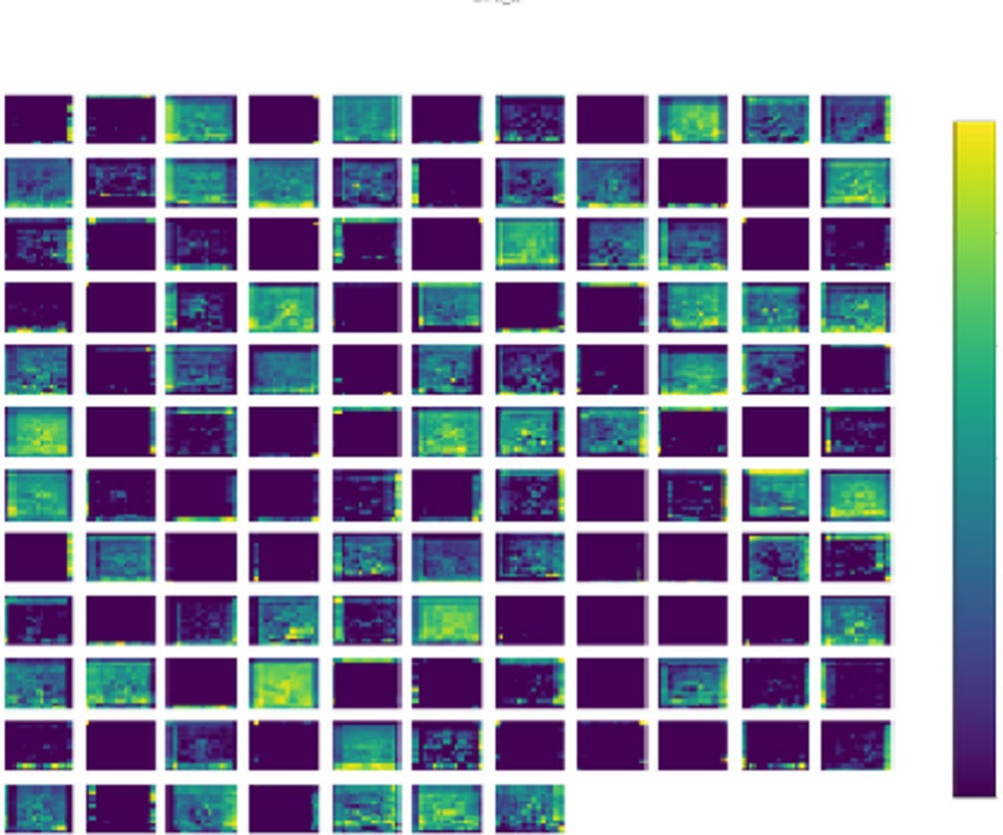
Feature visualization of the proposed model for the kidney disease dataset.

The inner layers of the proposed model produce visual representations of characteristics is shows in Fig. 8. These visually^43^ represented characteristics provide an in-depth examination and understanding of the patterns acquired by the machine learning model during the training phase. During the training process, every layer generates feature maps that highlight distinct patterns, functioning as a visual representation of the information obtained by the model. This study is crucial for understanding the specific parts of the input data that the model gives priority to, providing significant insights into its decision-making process.

### Evaluation metrics

The use of metrics for evaluation is essential in determining and evaluating the effectiveness and efficiency of the proposed method. The DBAR_Net model is evaluated using important metrics such as accuracy, precision, recall^44^, F1-score, and support for the classification of kidney disease into four classes based on CT images is seen in Table 4. The confusion matrix and the relative operating characteristic curve are two crucial tools used to assess the performance of the classification model at each layer. The support metric, although it does not have a defined formula, is used to show the frequency of occurrences in each class. The equations for calculating evaluation metrics^19^ are provided in equations (24) to (27).

24 $$\:\:\text{A}\text{c}\text{c}\text{u}\text{r}\text{a}\text{c}\text{y}:\text{A}\text{C}\text{C}=\:\frac{\text{T}\text{P}+\text{T}\text{N}}{\text{T}\text{P}+\text{F}\text{P}+\text{F}\text{N}+\text{T}\text{N}}$$

25 $$\:\:\:\text{P}\text{r}\text{e}\text{c}\text{i}\text{s}\text{i}\text{o}\text{n}:\text{P}\text{P}\text{V}=\:\frac{\text{T}\text{P}}{\text{T}\text{P}+\text{F}\text{P}}$$

26 $$\:\:\:\:\text{R}\text{e}\text{c}\text{a}\text{l}\text{l}:\text{R}\text{e}\text{c}\text{a}\text{l}\text{l}=\:\frac{\text{T}\text{P}}{\text{T}\text{P}+\text{F}\text{N}}$$

27 $$\:\:\:\:\text{F}1\:\text{S}\text{c}\text{o}\text{r}\text{e}:\text{F}1=2\:\text{X}\:\frac{\text{P}\text{P}\text{V}\text{X}\text{R}\text{e}\text{c}\text{a}\text{l}\text{l}}{\text{P}\text{P}\text{V}+\text{R}\text{e}\text{c}\text{a}\text{l}\text{l}}$$

**Table 4:** Evaluation metric of DBAR_Net and transfer learning model.

Model	Kidney class	Precision	Recall	F1-score	Accuracy (%)
VGG16	Cyst	0.81	0.75	0.78	97.50
	Stone	0.85	0.57	0.68	
	Kidney	0.56	0.98	0.72	
	Tumor	0.98	0.67	0.78	
VGG19	Cyst	0.98	0.99	0.98	97.44
	Stone	0.97	0.94	0.96	
	Kidney	0.97	0.97	0.97	
	Tumor	0.97	0.98	0.98	
ResNet50	Cyst	0.98	0.98	0.98	96.90
	Stone	0.96	0.94	0.95	
	Kidney	0.95	0.96	0.96	
	Tumor	0.97	0.98	0.98	
Xception	Cyst	0.98	0.98	0.98	97.26
	Stone	0.97	0.94	0.95	
	Kidney	0.96	0.97	0.97	
	Tumor	0.97	0.98	0.98	
Mobile Net	Cyst	0.99	0.98	0.98	97.22
	Stone	0.98	0.95	0.96	
	Kidney	0.97	0.98	0.98	
	Tumor	0.98	0.99	0.98	
InceptionV3	Cyst	0.99	0.97	0.98	96.90
	Stone	0.96	0.93	0.94	
	Kidney	0.95	0.97	0.96	
	Tumor	0.97	0.98	0.98	
EfficientNetB0	Cyst	0.96	0.99	0.97	97.26
	Stone	0.98	0.94	0.96	
	Kidney	0.97	0.98	0.97	
	Tumor	0.98	0.98	0.98	
Proposed DBAR_Net	Cyst	0.98	0.98	0.98	98.86
	Stone	0.98	0.94	0.96	
	Kidney	0.96	0.97	0.96	
	Tumor	0.97	0.98	0.97	

### Ablation study

For the purpose to determine the effect factor of various modules within the proposed DBAR_Net model which aims to classify five distinct categories of human activity an ablation research was conducted. The CT kidney Dataset from Kaggle is taken for this study. Identifying the five isolated activity classes is the aim, along with evaluating the accuracy, precision, recall, ROC curve, F1-score, and confusion matrix performance^40^. The suggested model incorporates convolved layer normalization blocks, a bottleneck attention module, and dilated convolved layer normalization, encompassing channel and spatial attention blocks. This setup is followed by a fully connected layer with flatten, dense, and dropout layers, which enhance interpretability and underscore the significance of input sequences in decision-making and debugging processes. The model achieves a total parameter count of 2,074,756. These parameters undergo iterative adjustments through optimization algorithms such as gradient descent to minimize the disparity between predicted outputs and actual targets during training. Similarly, the parameter values of the pre-trained model are also detailed in Table 6.

**Fig. 9:**
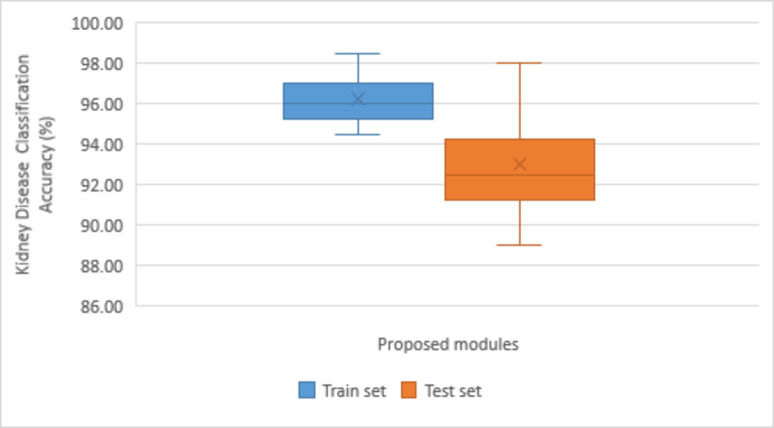
Graphical representation of performance comparison.

The first module of our DBAR_Net model, having the combination of convolved layer normalization blocks+ bottleneck attention module the accuracy reaching by 94.51% and 92.56% for the training and testing dataset. The second module convolved layer normalization blocks+ dilated convolved layer normalization reached the accuracy of 95.5% and 92.2%. The third module with convolved layer normalization blocks+ bottleneck attention module + dilated convolved layer normalization and the classification accuracy increased significantly to 96.5% and 89.02% respectively. The fourth module DBAR_Net model, the classification accuracy improved by reaching 98.51% and 98.02% for training and testing of four class kidney disease dataset. The result of these ablation study is shown in Table 5. The Fig. 9 explain the classification chart training and testing set for DBAR_Net model.

**Table 5:** Block-wise training and testing classification accuracy.

Blocks	Accuracy: kidney disease classification	
	Train set (%)	Test set (%)
$\:{\text{C}\text{L}\text{N}}_{\text{b}}+\:{\:\text{A}}_{\text{b}\text{a}\text{m}}$	94.51	92.56
$\:{\text{C}\text{L}\text{N}}_{\text{b}}\:+\:\text{D}{\text{C}\text{L}\text{N}}_{\text{b}}$	95.5	92.2
$\:{\text{C}\text{L}\text{N}}_{\text{b}}+\:{\:\text{A}}_{\text{b}\text{a}\text{m}}\:\:+\:\text{D}{\text{C}\text{L}\text{N}}_{\text{b}}$	96.5	89.02
Proposed DBAR_Net	99.32	98.86

Computational utilisation of the proposed model is measured using accuracy, model size (MB), total parameters, epochs running time (s), and time per step (ms) and is detailed in Table 6. DBAR_Net attains a compact model size of 7.91 MB with lesser parameters of 2,074,756 compared to other models. Each epoch was computed in achieving an accuracy of 98.8%.

**Table 6:** Computational efficiency of DBAR_Net model and transfer learning model.

Model	Testing accuracy (%)	Model size (MB)	Total parameter	Epochs running time (s)	Time per step (ms)
VGG16	97.5	56.13	14,714,688	1873	8000
VGG19	97.4	76.39	20,024,384	1538	7000
ResNet50	96.9	89.78	23,534,592	323	1000
Xception	97.2	79.37	20,806,952	477	2000
Mobile Net	97.2	8.61	2,257,984	95	425
InceptionV3	96.9	83.17	21,802,784	181	806
EfficientNetB0	97.2	15.45	4,049,564	414	2000
Proposed DBAR_Net	98.8	7.91	2,074,756	102	453

**Fig. 10:**
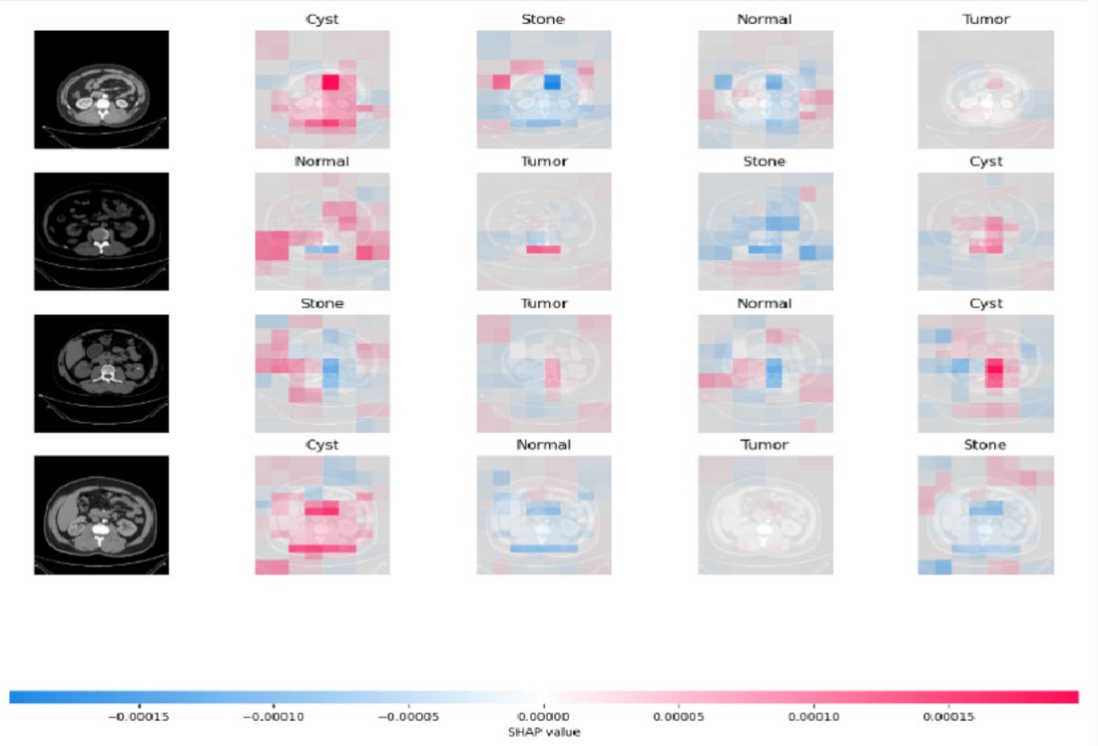
SHAP features of the proposed model.

**Fig. 11:**
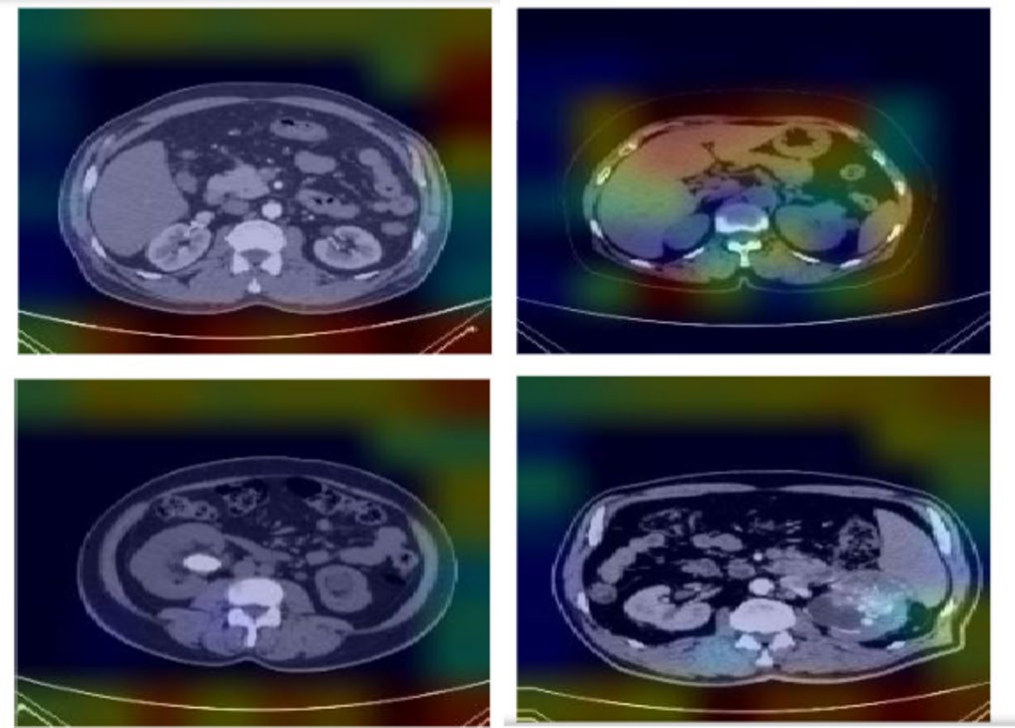
Grad-CAM features of the proposed model.

The interpretability of the proposed model is illustrated through Gradient-weighted Class Activation Mapping (Grad-CAM) and SHAP visualisation techniques. Grad-CAM^18^ highlights important regions in images that significantly impact the model’s predictions for every class. SHAP visualisation offer visual representation that indicate the importance of specific regions on the model’s decisions, enhancing its interpretability. Figures 10 and 11 shows the features obtained using SHAP^15^ and Grad-CAM showcasing the interpretability of the proposed work.

### Performance analysis of the proposed model using potato leaf disease dataset

**Fig. 12:**
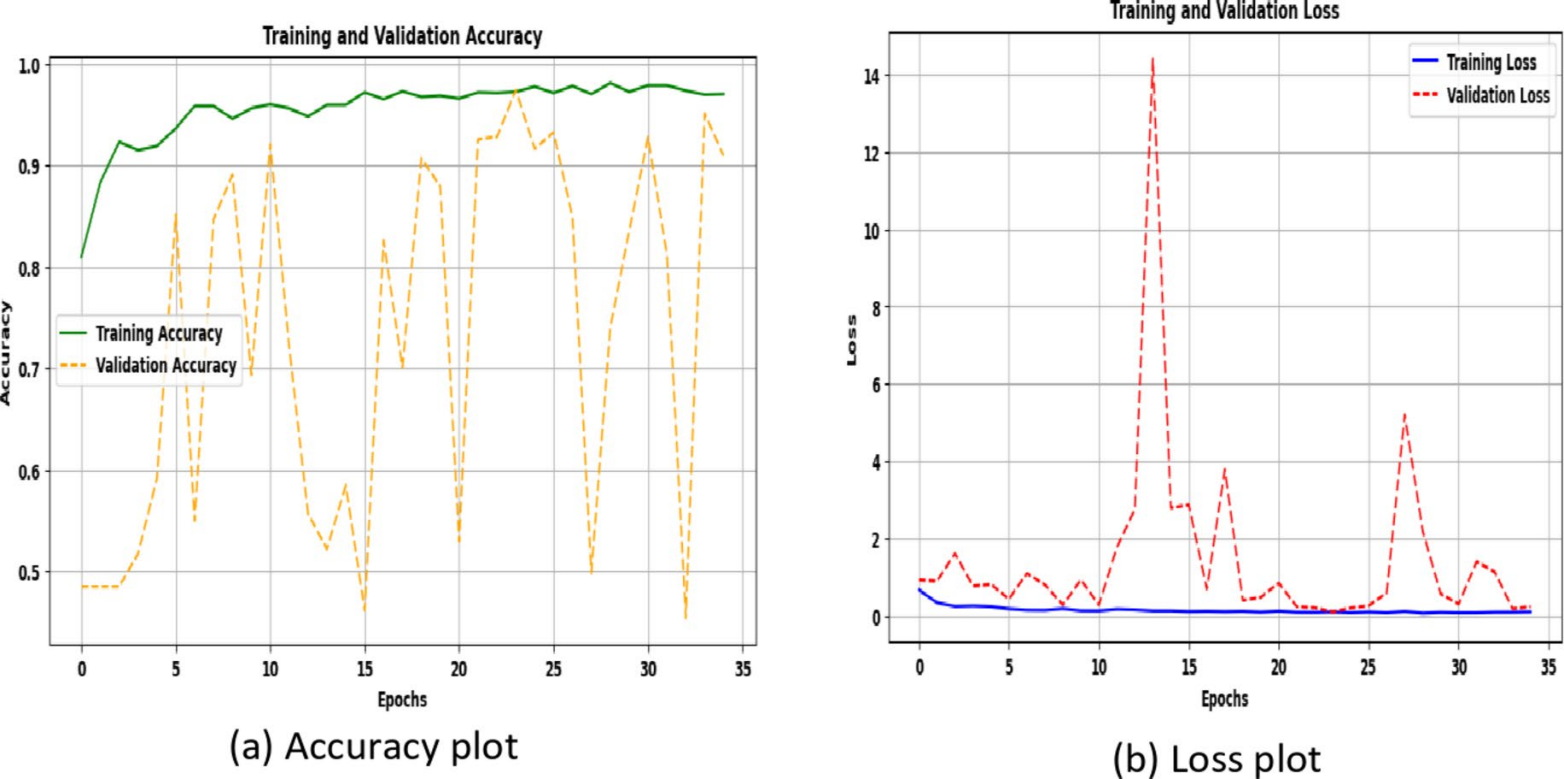
Training and validation curve of potato leaf disease dataset (**a**) accuracy plot (**b**) loss plot.

The scalability of the proposed DBAR_Net model is tested using potato leaf disease datasets^45^, demonstrating improved accuracy and robustness. Scalability refers to the ability of a model to maintain performance and efficiency as the size of the dataset or computational demands increase. Table 7; Fig. 12a and b detail the performance attained in terms of accuracy, as well as the training and validation loss.

**Table 7:** Performance metric of the proposed model for potato leaf disease dataset^45^.

Dataset	Classes	Dataset size	Input size	Epoch	Training time (per epochs)	Validation loss	Validation accuracy (%)
Potato leaf disease	Early Blight, Late Blight, Healthy	(1000, 152, 1000) **=** 2152	(256,256,3)	35	2 s	0.2286	90.9

### Comparison of the proposed DBAR_Net model with State_of_the_art architecture

Mehedi et al.^7^ segmented with UNet and utilized VGG16, with the latter achieving the highest accuracy. Jason et al.^6^ utilized Mask-RCNN and U-Net, attaining a test Dice score of 94.9. Mahmud et al.^12^ explored various models, with DenseAUXNet201 achieving 90.63% accuracy. Jhumka et al.^13^ employed xResNet 50 with the SHAP library, achieving 97% accuracy. Chen et al.^14^ evaluated AHDCNN and SVM using CT resulting versatile and improved features with 97.3% accuracy. Bhandari, Mohan, et al.^15^ employed XAI methods, including LIME, achieving a 98.88% accuracy through tenfold cross validation. Hadjiyski et al.^16^ the DLNN exhibited high accuracy in classifying the stages, achieving scores of 0.946 for Stage 1, 0.050 for Stage 2, 0.036 for Stage 3, and 0.031 for Stage 4 on the test CT scans images. Rajkumar et al.^17^ applied ANN and CNN, with CNN achieving 99.6% accuracy. Asif et al.^18^ used pre-trained VGG19 and naive Inception modules, achieving accuracies of 96.37% and 99.25%, respectively. Alzu’bi et al.^19^ evaluated VGG16 and ResNet50, with ResNet50 achieving 97.4% training and 96% validation accuracy. Hwang et al.^20^ used ResUNet, DenseUNet, Attention U-Net, and RBCA-Net, reaching accuracies of 77%, 79%, 81%, and 82% respectively.

Hsiao et al.^21^ utilized EfficientNet-B5, achieving various metrics including a Dice score of 91.50 and recall of 96.43. Islam et al.^22^ employed Vision Transformers and transfer learning models, with VGG16 and Swin Transformers reaching accuracies of 98.20% and 99.30%, respectively. Chaudhary et al.^23^ combined CNN + MLP and TabNet, achieving AUCs of 0.813 and 0.626, respectively. Zhang et al.^24^ utilized Coarse to Fine (C2F) segmentation, yielding 98.69% accuracy. Yildirim et al.^25^ analyzed the XResNet-50 model, achieving 96.82% accuracy in identifying stones from a private dataset. Da Cruz et al.^26^ employed AlexNet, achieving high segmentation metrics. Türk et al.^27^ evaluated various V-Net models, achieving an accuracy of 92.1%. Fuzhe et al.^28^ analyzed HMANN, reaching a 97.50% classification accuracy. Elmahy et al.^29^ evaluated using Graph Convolution Networks (GCN), Random Forest (RF), and Support Vector Machine (SVM), with GCN achieving 73% precision, improving to 82% with a two-weighed GCN and graph pooling. Table 8 presents a comparative examination between the Proposed DBAR_Net Model and the State_of_the_architecture.

**Table 8:** Key characteristics and performance of SOTA for kidney disease classification.

Ref	Algorithm	Method	Key characteristic	Accuracy (%)
^7^	UNet, SegNet, MobileNetV2, VGG16, and InceptionV3	Transfer Learning	Manual annotation of kidney CT images with expert validation and Grad-CAM visualisation for explainable model decisions	(VGG16) 99.48
^12^	DenseAUXNet201	Transfer Learning	Principal Component Analysis (PCA) to reduce dimensionality and feature selection techniques for optimisation.	90.63
^13^	XResNet 50 with SHAP	Transfer Learning	An early stopping function is employed to prevent overfitting.	97
^14^	AHDCNN and SVM	Deep Convolutional Neural Network	The model used a multi-layer deep belief network to identify irregular patterns in kidney disease data.	97.3
^16^	Deep Learning Neural Network, Inception V3	DLNN, Transfer Learning Model	The architecture balances depth and computational complexity.	91
^18^	VGG19 and Naive Inception	Transfer Learning Model	Additional layers were used to address the issues of vanishing gradients and overfitting.	99.25
^20^	RBCA-Net, ResUNet	Transfer Learning	Atrous Spatial Pyramid Pooling (ASPP) is utilised to extract spatial information.	82.79
^24^	Coarse to fine kidney segmentation	Convolutional Neural Network	Abnormality detection is performed using component analysis and a 2D convolutional neural network to correct the abnormal regions.	98.69
Proposed model	Dilated Bottleneck Attention-based Renal Network (DBAR-Net) Proposed Model	Multi-Feature Fusion Technique	Two fold convolved layer normalization blocks, dual bottleneck attention modules, dilated convolved layer normalisation block	98.86

## Conclusion and future work

The classification of kidney tumour, kidney stone, kidney cyst, and normal kidney in CT scans represents a significant advancement in the detection and diagnosis of renal disease. The “CT KIDNEY DATASET” is classified by two process one is proposed model and another one is transfer leaning model. The proposed DBAR_Net model includes multiple convolution layers, max pooling, and batch normalization with filter sizes of 32, 34, and 128 to prevent overfitting while learning hierarchy models. DBAR_Net elevates integration and stability through the utilization of custom dual-fold convolved layer normalization blocks $\:({\text{C}\text{L}\text{N}}_{\text{b}1}$ & $\:{\text{C}\text{L}\text{N}}_{\text{b}2})$. It amplifies comprehensibility and adaptability by prioritizing informative channel and spatial regions through the bottleneck attention module $\:{\:(\text{A}}_{\text{b}\text{a}\text{m}})$. Furthermore, the inclusion of dilated convolved layer normalization $\:({\text{D}\text{C}\text{L}\text{N}}_{\text{b}1}$) elevates spatial comprehension, augmenting semantic understanding of features. The acquired features undergo additive fusion, followed by global average pooling and layer normalization, effectively reducing spatial dimensions while preserving crucial information. As a result, this enhances the model’s ability to generalize and achieve improved results by addressing internal covariate shift. The DBAR_Net model has an accuracy of 98.51%. The second approach involves the use of some transfer learning models, such as VGG16, VGG19, ResNet50, EfficientNetB0, Inception V3, MobileNetV2, and Xception. This often involves the use of a pertained neural network as the foundation for a renal disease classification. Among these models, VGG16 achieves the best classification accuracy at 97.50%, when compare the proposed model with transfer learning model proposed achieve the best classification accuracy which allows for parallelization of computation. In between the process data augmentation occurs with some hyper parameter tuning process in which performance can be determined by evaluation metric of precision, recall, and F1-score in classification tasks, providing a deeper understanding of a model’s performance beyond simple accuracy. Continuous validation and benchmarking against state-of-the-art techniques are vital to ensure competitiveness and effectiveness in clinical decision-making for renal disease diagnosis. Overall, the suggested model assists medical examiners in making more accurate clinical decisions when diagnosing renal diseases. Future research could focus on refining DBAR_Net architecture with advanced CNN features, integrating multi-modal data and optimizing transfer learning strategies for improved renal disease classification accuracy and clinical applicability.

## Data Availability

The Dataset is publicly available “CT KIDNEY DATASET”, the link to access the data “ https://www.kaggle.com/datasets/nazmul0087/ct-kidney-dataset-normal-cyst-tumor-and-stone”.
